# ForestFoodKG: A Structured Dataset and Knowledge Graph for Forest Food Taxonomy and Nutrition

**DOI:** 10.3390/foods14244186

**Published:** 2025-12-05

**Authors:** Rongen Yan, Zhidan Chen, Shengqi Zhou, Guoxing Niu, Yan Li, Zehui Liu, Jun Wang, Xinwan Wu, Qi Luo, Yibin Zhou, Yanting Jin, Keyan Liu, Weilong Yuan, Jingyi Xu, Fu Xu

**Affiliations:** 1 School of Information Science and Technology, Beijing Forestry University, No. 35, Qinghua East Road, Beijing 100083, China; reyan2023@bjfu.edu.cn (R.Y.); liyan2003@bjfu.edu.cn (Y.L.); jyt0723@bjfu.edu.cn (Y.J.); 2 Engineering Research Center for Forestry-Oriented Intelligent Information Processing of National Forestry and Grassland Administration, Beijing 100083, China; 3 Hebei Key Laboratory of Smart National Park, Beijing 100083, China; 4 School of Landscape Architecture, Beijing Forestry University, Beijing 100083, China; zhoushengqi031@bjfu.edu.cn (S.Z.); alwaysu0112@bjfu.edu.cn (Y.Z.); lky220201616@bjfu.edu.cn (K.L.); ywl610985@bjfu.edu.cn (W.Y.); 5 School of Economics & Management, Beijing Forestry University, Beijing 100083, China; liuzehui1211@bjfu.edu.cn; 6 College of Forestry, Beijing Forestry University, Beijing 100083, China; wangjun1704@bjfu.edu.cn (J.W.);; 7 School of Foreign Languages, Beijing Forestry University, Beijing 100083, China; xjy18600235215@bjfu.edu.cn

**Keywords:** forest food, taxonomy, nutritional information, knowledge graph, dataset construction

## Abstract

Forest foods play a vital role in enhancing dietary diversity, human health, and the sustainable use of forest ecosystems. However, structured and machine-readable resources that systematically describe their taxonomic and nutritional attributes remain scarce. To fill this gap, we introduce ForestFoodKG, a comprehensive resource that integrates taxonomic hierarchy and nutritional composition of 1191 forest food items. The resource consists of two components—(i) the ForestFoodKG dataset, containing standardized taxonomic and nutritional records across seven biological levels, and (ii) the ForestFoodKG Knowledge Graph (ForestFoodKG-KG), which semantically links forest food entities using named entity recognition and relation extraction. The constructed graph comprises 4492 entities and 14,130 semantic relations, providing a structured foundation for intelligent querying, nutrition analytics, and ecological informatics. All data were manually verified and made publicly available in CSV format on GitHub. ForestFoodKG serves as the first structured knowledge base for forest foods, promoting data-driven research in nutrition science, sustainable forestry, and knowledge-based decision-making.

## 1. Introduction

Global food systems face an increasing challenge of nutritional homogenization. Although over 250,000 plant species are known to be edible, more than 75% of the world’s food supply relies on only a few dozen species [1,2,3,4]. This imbalance poses risks to food security, biodiversity, and human nutrition. Forest ecosystems, which cover approximately one-third of the Earth’s land area, represent a vast but underexplored source of edible resources, including fruits, nuts, fungi, and wild vegetables [5,6,7]. These resources not only contribute to dietary diversification and micronutrient intake but also support local livelihoods and ecological sustainability [8,9,10].

Despite their ecological and nutritional value, forest foods remain poorly represented in structured digital resources. Existing information is scattered across scientific papers, field reports, and websites, often using inconsistent taxonomic and nutritional descriptions [11,12]. Such heterogeneity hinders the development of data-driven applications in smart agriculture, food informatics, and ecological knowledge management [13].

In recent years, knowledge graphs (KGs) have emerged as a powerful framework for integrating heterogeneous data and enabling intelligent reasoning in domains such as crop disease prediction, food traceability, and nutrition analytics [14,15,16]. However, to our knowledge, no existing KG focuses specifically on forest foods, leaving a significant gap between ecological data and computational nutrition analysis.

To bridge this gap, we introduce ForestFoodKG, a two-part resource comprising (i) the ForestFoodKG dataset, a curated collection of 1191 forest food items annotated with hierarchical taxonomic and nutritional attributes, and (ii) the ForestFoodKG knowledge graph (ForestFoodKG-KG), a semantic graph constructed using named entity recognition (NER) and relation extraction techniques. The dataset includes seven-level taxonomy (from kingdom to species) and key nutritional indicators such as proteins, fats, carbohydrates, vitamins, and minerals. Here, the ForestFoodKG dataset comprises unstructured text on forest food names, classification information, and nutritional components crawled and compiled from online resources, while ForestFoodKG-KG is a knowledge graph constructed on this basis through information extraction to obtain structured triplets and built using Neo4j. To build ForestFoodKG-KG, we manually annotated 400 domain-specific sentences in BIO format and trained a BERT-BiLSTM-CRF model, which achieved an F1-score of 91.35% in NER. We then applied both rule-based and model-driven relation extraction methods to construct a Neo4j-based knowledge graph containing 4492 entities and 14,130 relations.

The contributions of this paper are summarized as follows.

(1) A Novel Dataset: We introduce the ForestFoodKG dataset, a curated collection of 1191 forest food items annotated with seven-level taxonomic classifications and nutritional components, addressing a critical data gap in the field.

(2) An Effective Pipeline: We propose a knowledge extraction framework for the forest food domain, which involves manual BIO annotation and a high-performance model, demonstrating robust capability in identifying domain-specific entities.

(3) A Functional Knowledge Graph: We construct the ForestFoodKG-KG using Neo4j. This graph integrates 4492 entities and 14,130 labeled edges, providing a semantic foundation that supports advanced applications.

The remainder of this paper is organized as follows. Section 2 describes the data collection, annotation, and graph construction methods. Section 3 presents experimental results and analysis. Section 4 concludes the study and outlines future research directions.

## 2. Methodology

**Figure 1:**
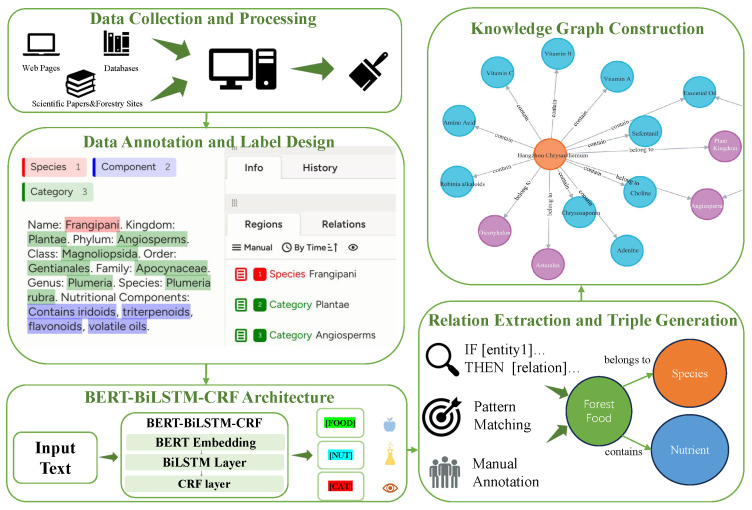
Workflow of the ForestFoodKG construction process. The diagram illustrates the pipeline from raw data collection to the final knowledge graph.

This section describes the workflow for constructing the ForestFoodKG dataset and knowledge graph. The process involves four major steps: (i) data collection and standardization, (ii) manual annotation for NER, (iii) model-based entity and relation extraction, and (iv) graph construction and validation. The entire workflow is shown in Figure 1.

### 2.1. Sources of Data on Forest Foods

To ensure that the collected dataset is comprehensive in breadth and profound in its professional scope, we meticulously selected and integrated valuable information resources from numerous renowned authoritative institutions, international organizations, and esteemed journals. This dataset is rich in content, encompassing various fields, including medicinal forest plants, forest foods, forest oils, forest fruits, forest beverages, forest medicines, forest bee products, forest spices, forest nuts, forest meats, and forest teas. Here, we present a partial list of data sources, their corresponding websites, and the scope of the data they cover in the form of a table, as shown in Table 1.

### 2.2. Data Collection and Processing

In the collection and processing of forest food-related data, we employed a variety of methods to gather information from diverse sources and construct a structured resource, providing a solid foundation for subsequent research and applications. The data were primarily obtained from online sources and scientific literature.

We performed standardization on all fields (including Kingdom, Phylum, Class, Order, Family, Genus, Species, and Nutrition Information) by removing duplicate entries, missing values, extraneous spaces, line breaks, and non-standard characters, thereby ensuring dataset consistency. The total number of records before cleaning was 1469, while the number of valid records after cleaning was 1191. Regarding missing value processing, Table 2 provides a detailed comparison of the quantities before and after cleaning. Taking the Species field as an example, there were 207 missing records before cleaning, accounting for 14.09% of the original data; after cleaning, the number of missing records was reduced to 19, representing only 1.60% of the cleaned dataset.

A comparison of the overall metrics before and after data cleaning is presented in Table 3. After cleaning, the total number of records decreased from 1469 to 1191, and the duplicate rate was reduced from 12.87% to 6.89%. The vocabulary size and total tokens for nutrient information were also refined, while control character noise was completely eliminated. This reduction was primarily attributed to two key actions: first, the removal of duplicate entries to ensure data uniqueness; second, and more critically, the exclusion of records for which essential information (such as species name or detailed nutritional profiles) could not be verified or located across authoritative public databases and scientific literature after an exhaustive search. These unverifiable records were deemed to lack the necessary integrity for inclusion in a high-quality knowledge graph and were consequently dropped.

### 2.3. Data Annotation and Label Design

**Figure 2:**
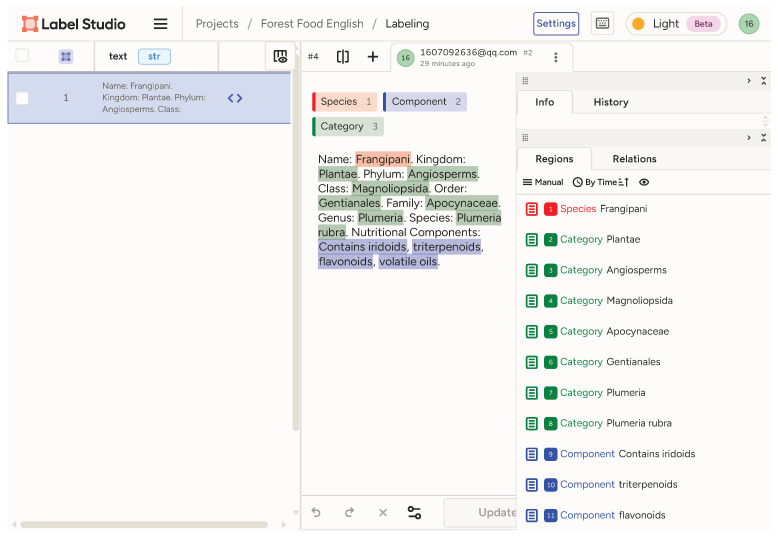
Example diagram of entity annotation using the Label studio tool.

Building upon the foundational dataset acquired, we pursued high-fidelity entity recognition employing the BIO  [17] (Begin, Inside, Outside) labeling scheme. We performed manual annotation using the Label Studio tool, with an example illustration shown in the Figure 2. The annotation work was carried out by a team of domain specialists with backgrounds in forestry and food science, utilizing the Label Studio platform.

This approach facilitated the meticulous manual annotation of 400 sentences centered on forest food. We collected a total of 400 sentences, which were ultimately processed into 1191 individual data records. Our annotation concentrated on identifying three pivotal entity types: FOOD, representing forest food names; NUT, encapsulating nutritional components such as vitamins and minerals; and CAT, covering taxonomic levels ranging from kingdom to species. Each token was meticulously labeled as B-X, I-X, or O, ensuring precise entity delineation in the corpus (Table 4).

The annotated sentences were serialized into JSON format and subdivided into training (70%), validation (20%), and test (10%) datasets, constructing a robust foundation for model training and evaluation.

### 2.4. BERT-BiLSTM-CRF Architecture

To address sequence labeling for named entity recognition with precision, we deployed an advanced BERT-BiLSTM-CRF architecture [18]. This hybrid model synergistically combines the strengths of contextual embeddings, sequential modeling, and structured prediction to enhance recognition capability in domains requiring specialized terminology.

The architecture begins with a BERT encoder, which generates contextualized embeddings for each token in the input sequence. Unlike traditional word embeddings that assign fixed representations regardless of context, BERT produces dynamic embeddings that capture nuanced semantic and syntactic information based on surrounding words. This is achieved through a multi-layer bidirectional transformer architecture that processes text in both directions simultaneously, allowing each token representation to be informed by its complete contextual environment.

The contextual embeddings from BERT are then fed into a Bidirectional Long Short-Term Memory (BiLSTM) network. The BiLSTM layer captures sequential dependencies in both forward and backward directions, modeling how entity mentions typically exhibit patterns and constraints across token sequences. This bidirectional processing enables the model to consider both preceding and succeeding context when interpreting each token, which is particularly valuable for identifying entity boundaries in the forest food domain.

Finally, a Conditional Random Field (CRF) layer performs structured decoding of the entire output sequence. Rather than predicting each tag independently, the CRF layer considers the compatibility between adjacent tags and learns global transition patterns between them. This ensures that the predicted tag sequence follows valid patterns—for instance, that an “I-FOOD” tag typically follows a “B-FOOD” tag rather than an “O” tag—thereby producing more coherent and linguistically plausible entity annotations.

This three-component architecture leverages BERT’s contextual understanding, BiLSTM’s sequence modeling capabilities, and CRF’s structured prediction to achieve robust entity recognition performance in our specialized domain.

### 2.5. Relation Extraction and Triple Generation

The construction of high-quality semantic relations is essential for building a meaningful knowledge graph. Our approach combines rule-based pattern matching with context-aware scoring to extract relations with high confidence.

For taxonomic relationships, particularly the FOOD-belongs_to-CAT relation, we employed rule-based extraction using carefully designed regular expressions and syntactic patterns. This method effectively captures explicit hierarchical statements found in the text, such as the example “Pine nut belongs to the family Pinaceae”, which is directly mapped to the triple (pine nut, belongs_to, Pinaceae).

To handle the more nuanced expression of nutritional relationships, we developed a shallow semantic scoring framework. This approach evaluates candidate entity pairs using a scoring function that considers both the proximity between entities and the presence of relational indicators. The scoring function is defined as:

(1) $$\mathrm{Score}\left(e_{i},e_{j}\right)=\frac{1}{1+\mathrm{dist}(e_{i},e_{j})}\cdot I\left(\mathrm{relation}\;\mathrm{keyword}\right)$$

where $\mathrm{dist}(e_{i},e_{j})$ represents the token distance between entities and I is an indicator function for relational keywords. High-scoring pairs are confidently mapped to (FOOD, contains, NUT) triples, enabling efficient relation extraction without deep syntactic analysis. To ensure accuracy, all extracted relationships underwent a rigorous review process by a panel of three food science domain experts. This multi-expert workflow focused on verifying and correcting erroneous relationships through iterative rounds of annotation. Given that the correction process involved dynamic, collaborative discussions rather than independent parallel annotations, we did not calculate a quantitative error rate. Instead, we prioritized achieving a expert-validated, high-quality set of relationships for the knowledge graph.

The extracted triples follow the formal structure $(e_{h},r,e_{t})\in G$, where $e_{h}$, *r*, and $e_{t}$ represent the head entity, relation type, and tail entity respectively. These triples were systematically formatted in both CSV and JSON formats to ensure interoperability and facilitate seamless integration into the Neo4j graph database.

### 2.6. Knowledge Graph Construction

The ForestFoodKG knowledge graph is implemented using Neo4j [19], an open-source graph database management system specifically designed to handle interconnected data. The graph is generated by populating Neo4j with triples, whose entities are extracted from the ForestFoodKG dataset. Unlike traditional relational databases that rely on tabular structures, Neo4j represents information natively as a graph—composed of nodes, edges, and properties—making it particularly suitable for modeling complex domain relationships.

At the core of our implementation is a graph model in which nodes represent key entity types such as forest foods (FOOD), nutrients (NUT), and taxonomic categories (CAT). These nodes are interconnected through semantically meaningful edges, including belongs_to for taxonomic hierarchies and contains for nutritional associations. Each node and relationship can store properties, enabling the attachment of rich metadata essential for a domain-specific knowledge graph.

Neo4j’s query language, Cypher, provides an intuitive and expressive means to explore the graph. It supports complex traversals, pattern matching, and path analysis, allowing users to efficiently query nutritional profiles of specific foods or trace taxonomic lineages across multiple biological ranks. This capability is vital for supporting interactive exploration and analytical queries within the ForestFoodKG framework.

In the context of ForestFoodKG, Neo4j offers significant advantages in managing the intricate web of taxonomic and nutritional relationships. Its native graph architecture enables efficient traversal of connections between entities, facilitating the discovery of indirect associations and supporting advanced semantic queries. The current graph instantiation comprises 4492 nodes and 14,130 edges, forming a scalable foundation that can be extended as new data becomes available.

**Figure 3:**
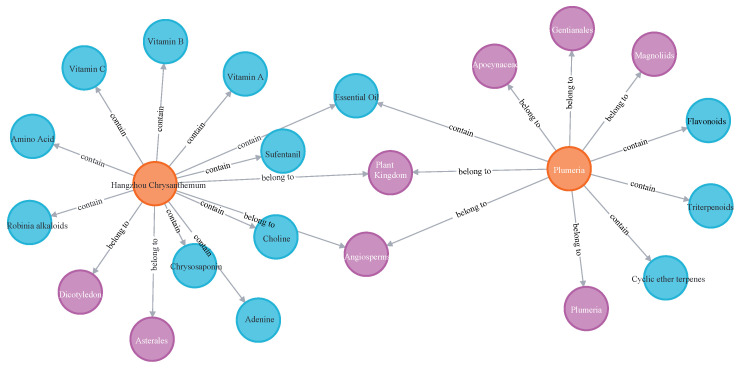
Knowledge graph for forest food.The spheres are labeled with the names of the key items they represent, and the grey arrows describe how they are related. The central item, “Forest Food” (in orange), serves as the main category. It is directly linked to specific “Nutrients” (blue spheres) and broader “Plant Categories” (purple spheres).

The resulting knowledge graph is both semantically rich and visually explorable. Figure 3 provides a structural overview of the ontology, illustrating the interconnected nature of forest food entities, their classifications, and nutritional attributes. By leveraging Neo4j, ForestFoodKG not only represents domain knowledge effectively but also enables intuitive querying and visualization, thereby advancing research capabilities in forest food informatics.

### 2.7. Evaluation Metrics

Model performance was quantified using standard information extraction metrics: precision (Equation (2)), recall (Equation (3)), and their harmonic mean, the F1-score (Equation (4)).

(2) $$\begin{array}{cc} \mathrm{Precision} & =\frac{TP}{TP+FP} \end{array}$$

(3) $$\begin{array}{cc} \mathrm{Recall} & =\frac{TP}{TP+FN} \end{array}$$

(4) $$\begin{array}{cc} F1\text{-}\mathrm{score} & =\frac{2\cdot \mathrm{Precision}\cdot \mathrm{Recall}}{\mathrm{Precision}+\mathrm{Recall}} \end{array}$$

where TP, FP, and FN represent true positives, false positives, and false negatives, respectively.

To ensure the robustness and generalizability of our findings, we conducted extensive experiments exploring diverse hyperparameter configurations, including variations in learning rate, batch size, and hidden layer dimensions. Furthermore, we employed k-fold cross-validation (with k = 5) to assess model stability across different data partitions. This comprehensive evaluation strategy ensures that the reported performance metrics reflect consistent model behavior rather than optimistically biased results from a single training instance.

## 3. Experiments and Discussion

### 3.1. Dataset

**Figure 4:**
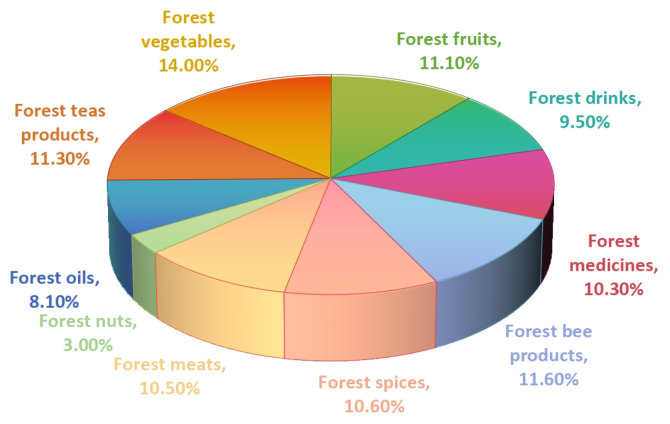
Distribution and proportion of forest food categories pie chart.This distribution highlights the diverse yet uneven nature of documented forest food resources.

After successfully obtaining the ForestFoodKG dataset, we conduct a comprehensive and in-depth statistical analysis of it. As shown in Figure 4, the ForestFoodKG dataset encompasses ten categories, including forest vegetables, forest fruits, forest teas, forest bee products, forest meats, forest spices, and forest medicines, providing a comprehensive overview of forest food resources. Despite the rich variety and extensive coverage of categories, the structure of the dataset exhibits a certain degree of imbalance. Among these categories, forest vegetables rank first with a proportion of 14.00%, indicating their significant position within forest food resources. Following closely are forest teas, forest fruits, Forest bee products, forest spices, forest meats, and forest medicines, each with a share exceeding 10%, collectively forming an important pillar of the forest food industry. In contrast, forest nuts have the lowest proportion among all categories, revealing their relative scarcity in forest food resources.

In addition, we conducted a detailed taxonomic analysis of the collected forest food resources, encompassing various levels of classification, including kingdom, phylum, class, order, family, genus, and species. In this study, we conduct a systematic taxonomic analysis of the collected forest food resources, covering all classification levels from kingdom to species, including kingdom, phylum, class, order, family, genus, and species. According to the ForestFoodKG records, the forest food resources involve three major biological kingdoms. Among these, the plant kingdom holds an absolute dominance, with a total of 1011 species, accounting for 84.9% of the entire dataset. In contrast, the fungal kingdom represents the smallest proportion, with only 16 species, accounting for 1.3%. The animal kingdom includes 164 species, making up 14.8% of the dataset.

In the ForestFoodKG, the records encompass 24 different biological phyla. Among them, the phylum Angiosperms ranks first with a frequency of 941 occurrences, significantly higher than that of other phyla. Following closely is the phylum Chordata, with 113 occurrences. The remaining phyla have relatively low frequencies in the dataset. We conduct a statistical analysis of the distribution of phyla, and the results show that the number of species in Angiosperms and Chordata is significantly higher than in other groups. This finding indicates that Angiosperms and Chordata dominate the classification of forest foods.

The dataset is categorized into 39 different biological classes, ranging from plant classes such as Magnoliopsida and Pinopsida to fungal classes like Basidiomycetes and Agaricomycetes. Among these classes, Magnoliopsida and Dicotyledoneae are particularly prominent, occupying a significant proportion. These two classes play a key role in the diversity of forest foods, highlighting the richness and complexity of the plant kingdom. To more clearly depict the distribution characteristics of these classes, we further describe the distribution of several major classes.

The ForestFoodKG records a total of 167 different biological orders, encompassing various classifications such as Gentianales, Asterales, Erythropalales, Cucurbitales, Saxifragales, and Campanulales, among others. These classifications reflect the wide distribution of forest foods within biodiversity.

### 3.2. Data Records

The ForestFoodKG compiles 1191 detailed records, each presented accurately in Chinese, with precise separation by blank lines to ensure high readability. Given the scarcity of information on forest foods, this compilation represents the largest and most unique dataset of its kind currently known. An overview of the relevant fields and content can be found in Table 5.

Table 6 provides examples of two records from the dataset.

The taxonomy of forest foods and their rich nutritional component information provide extensive application potential for data modeling in artificial intelligence. These data can be utilized for personalized health analysis and nutritional recommendations, monitoring food safety risks, assessing medicinal value, analyzing market trends, optimizing agricultural and forestry management, and supporting the evaluation of ecosystem services. Furthermore, this information can aid in the development of educational tools to enhance public awareness and consumption of forest foods, thereby promoting sustainable development and the rational utilization of resources.

### 3.3. Experimental Setup

All models were trained on the manually annotated ForestFood dataset using a consistent experimental protocol. For the BERT-Softmax and BERT-BiLSTM-Softmax architectures, we employed the standard cross-entropy loss function, while the BERT-BiLSTM-CRF model was optimized using CRF loss, which explicitly models label transition constraints. To mitigate overfitting, we implemented an early stopping mechanism based on validation set performance, with patience set to 5 epochs. The training configuration utilized a fixed batch size of 32 across all experiments, with models trained for a maximum of 20 epochs. Hyperparameter optimization, particularly for the learning rate, was conducted through systematic grid search to ensure optimal performance.

The specific hyperparameter configurations employed in our BERT-BiLSTM-CRF model are detailed in Table 7, ensuring full reproducibility of our experimental results.

### 3.4. Results and Discussion

The foundation for constructing a high-quality knowledge graph lies in the accurate identification of entities from unstructured text, which represents the core objective of the NER task. As the initial and most critical step in the pipeline, the performance of the NER model directly determines the completeness and correctness of all subsequent entities and relationships in ForestFoodKG-KG. To systematically evaluate entity recognition capabilities in the forest food domain, we compared three mainstream architectures: BERT-Softmax, BERT-BiLSTM-Softmax, and BERT-BiLSTM-CRF. After 20 training epochs, the performance metrics of each model on the test set are presented in Table 8.

Experimental results demonstrate that BERT-BiLSTM-Softmax achieved the best performance with an F1-score of 0.9135, while BERT-BiLSTM-CRF attained the highest recall rate of 0.8944. This finding challenges the conventional preference for CRF-based architectures in NER tasks, suggesting that in specialized domains with limited training data, the additional complexity introduced by CRF layers may not yield proportional performance benefits. Notably, all BiLSTM-enhanced models significantly outperformed the baseline BERT-Softmax, confirming the importance of sequence modeling for handling complex entity structures in the forest food domain.

This study provides important references for knowledge graph construction in ecological and agricultural domains. The experimental results reveal a crucial trade-off in model selection: BERT-BiLSTM-Softmax offers more balanced overall performance, while BERT-BiLSTM-CRF demonstrates advantages in entity coverage. We hypothesize that the CRF architecture might realize its full potential with larger training datasets, representing a key direction for future investigation. This research validates the feasibility of constructing domain-specific knowledge graphs with limited annotated resources, establishing a solid foundation for downstream applications such as dietary recommendation systems and ecological research.

## 4. Conclusions and Future Work

This study constructed ForestFoodKG, a structured dataset and knowledge graph that systematically integrates the taxonomic hierarchy and nutritional attributes of forest-derived foods. By combining expert-curated data with machine learning–based entity and relation extraction, the proposed framework transforms fragmented textual information into a semantically organized resource. ForestFoodKG serves as the first domain-specific knowledge base for forest foods, providing standardized, machine-readable representations that support intelligent querying, nutritional analysis, and cross-disciplinary research. The results demonstrate that integrating biological taxonomy with nutrient data offers a feasible and effective approach to advancing digital resource management in forest food research.

Building upon this foundation, future research will focus on three major directions. First, the dataset will be expanded and enriched through the incorporation of additional sources such as biodiversity inventories, metabolomic databases, and regional forestry records, thereby improving data coverage and granularity. Second, efforts will be made toward cross-domain integration, linking ForestFoodKG with other open agricultural and nutritional knowledge graphs (e.g., FoodKG, AgroKG) to enhance semantic interoperability and support broader ecological–nutritional analyses. Finally, we plan to develop interactive visualization and application systems based on the ForestFoodKG-KG, enabling practical use in personalized nutrition recommendation, forest product traceability, and ecological education. These extensions will further strengthen the role of ForestFoodKG as a sustainable digital infrastructure for food informatics and ecological knowledge management.

## Figures and Tables

**Table 1:** Overview of main data sources.

Data Source	Link	Scope
USDA National Nutrient Database	https://catalog.data.gov/dataset/ (accessed on 14 July 2025)	Food and nutrient composition data
iPlant Platform	https://www.iplant.cn (accessed on 5 January 2025)	Botanical data
Plant Science Data Center	https://www.plantplus.cn/doi/10.12282/plantdata.1390 (accessed on 17 February 2025)	Plant science data
China Forestry Network	http://www.isenlin.cn/species.html (accessed on 23 March 2025)	Forestry species data
China Forest Food Website	https://www.forestfood.com.cn/ (accessed on 12 April 2025)	Forest foods
Chinese Biodiversity Database	https://species.sciencereading.cn/ (accessed on 27 May 2025)	Biodiversity information
Guangdong Agricultural Sciences	https://gdnykx.gdaas.cn/ (accessed on 3 June 2025)	Forestry and agricultural studies
Chinese Journal of Tropical Crops	http://www.rdzwxb.com/EN/current (accessed on 15 June 2025)	Tropical crops research
Hubei Forestry Science and Technology	http://www.inforhubei.com/hblykj/home/ (accessed on 7 July 2025)	Forestry technology studies
Journal of Nanjing Forestry University	http://nldxb.njfu.edu.cn/CN/1000-2006/home.shtml (accessed on 19 July 2025)	Forestry and ecology
Anhui Agricultural Sciences	https://www.ahnxtb.cn/EN/home (accessed on 2 February 2025)	Comprehensive agricultural and forestry research
Science and Technology of Food Industry	https://www.spgykj.com/ (accessed on 28 April 2025)	Food science and technology
Flavor Fragrance Cosmetics	https://www.fda.gov/ (accessed on 28 April 2025)	Fragrances, flavors, and cosmetics studies
Journal of Sichuan Agricultural University	https://journal.scau.edu.cn/indexen.htm (accessed on 9 May 2025)	Comprehensive agricultural research
Food Science	https://foodscience.com/ (accessed on 9 May 2025)	Food nutrition research
China Pharmaceuticals	http://tg.zhongguoyaoye023.com/default.aspx (accessed on 9 May 2025)	Pharmaceuticals and forest-derived medicines

**Table 2:** Overall metrics of data cleaning.

Metric	Raw Data	Cleaned Data
Total Records	1469	1191
Duplicate Rate	12.87%	6.89%
Vocabulary Size (Nutrient Info)	4083	3475
Total Tokens (Nutrient Info)	8995	7502
Noise Rate (control characters)	0.07%	0.00%

**Table 3:** Overall missing values statistics.

Field	Missing Values Before Cleaning	Missing Values After Cleaning
Kingdom	1	1
Phylum	3	1
Class	4	1
Order	4	1
Family	3	0
Genus	7	2
Species	207	19
Nutritional component	4	4

**Table 4:** Entity types and label format.

Label	Description
B-FOOD/I-FOOD	Start/Interior of forest food name
B-NUT/I-NUT	Start/Interior of nutritional component
B-CAT/I-CAT	Start/Interior of taxonomic category
O	Outside any defined entity

**Table 5:** Comprehensive overview of the ForestFoodKG dataset fields.

Field Name	Description
**name**	The Chinese name of forest foods.
**kingdom**	To which kingdom do forest foods belong in biological taxonomy? The ForestFoodKG consists of three distinct kingdoms: the plant kingdom, the animal kingdom, and the fungal kingdom.
**phylum**	To which phylum do forest foods belong in biological taxonomy? The ForestFoodKG includes a total of 24 different phyla, such as Angiosperms, Gymnosperms, Ascomycota, Pteridophyta, Basidiomycota, and others.
**class**	To which class do forest foods belong in biological taxonomy? The ForestFoodKG includes a total of 39 different classes, such as Magnoliopsida, Dicotyledons, Coniferopsida, and Monocotyledons.
**order**	To which order do forest foods belong in biological taxonomy? The ForestFoodKG includes a total of 167 different orders, such as Gentianales, Asterales, Lamiales, and Cucurbitales.
**family**	To which family do forest foods belong in biological taxonomy? The ForestFoodKG includes a total of 270 different families, such as Apocynaceae, Asteraceae, Caprifoliaceae, and Rubiaceae.
**genus**	To which genus do forest foods belong in biological taxonomy? The ForestFoodKG includes a total of 638 different genera, such as Plumeria, Chrysanthemum, Lonicera, Coffea, Siraitia, and Paeonia.
**species**	To which species do forest foods belong in biological taxonomy? There are a total of 1191 different forest foods.
**nutritional component**	A textual description of the nutritional components of forest foods. For example, polysaccharides, flavonoids, and minerals such as iron, zinc, copper, manganese, and selenium.

**Table 6:** Two examples from the ForestFoodKG dataset.

Name	Giant Himalayan Lily	Phyllostachys Pubescens
**kingdom**	Plant kingdom	Plant kingdom
**phylum**	Angiosperm phylum	Vascular Plant phylum
**class**	Magnolia class	Magnolia class
**order**	Liliales	Poales
**family**	Liliaceae	Poaceae
**genus**	Lilium genus	Phyllostachys genus
**species**	*C. giganteum*	*Phyllostachys edulis*
**nutritional composition**	The bulbs of the Giant Himalayan Lily are rich in starch, dietary fiber, as well as vitamin C and minerals such as potassium and magnesium.	The young shoots are rich in protein, dietary fiber, vitamin C, B vitamins, and minerals such as potassium, calcium, and magnesium.
**figure**	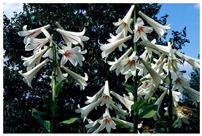	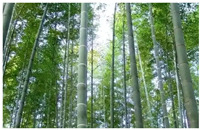

**Table 7:** BERT-BiLSTM-CRF model parameter settings.

Parameter Name	Description	Value
epoches	Number of epochs	20
batch_size	Batch size	32
hidden_size	BERT hidden layer size	768
Bert-lr	BERT layer learning rate	$3\times 10^{-5}$
CRF-lr	CRF layer learning rate	0.001
BiLSTM-lr	BiLSTM layer learning rate	0.0001
BiLSTM-hidden_size	BiLSTM layer hidden size	256
Dropout	Dropout rate	0.1

**Table 8:** Comparative performance of NER models on the ForestFood dataset. The bolded part indicates the best effect.

Model	Precision	Recall	F1-Score
BERT-Softmax	0.8985	0.8917	0.8946
BERT-BiLSTM-Softmax	**0.9440**	0.8853	**0.9135**
BERT-BiLSTM-CRF	0.8895	**0.8944**	0.8918

## Data Availability

The ForestFoodKG dataset is now officially available on the github (https://github.com/dadadaray/FTAND (accessed on 19 November 2025)) platform and can be accessed in a CSV file format by the public.

## References

[1] Weiss A.S., Niedermeier L.S., von Strempel A., Burrichter A.G., Ring D., Meng C., Kleigrewe K., Lincetto C., Hübner J., Stecher B. (2023). Nutritional and host environments determine community ecology and keystone species in a synthetic gut bacterial community. Nat. Commun..

[2] Imathiu S. (2021). Neglected and underutilized cultivated crops with respect to indigenous African leafy vegetables for food and nutrition security. J. Food Secur..

[3] Maitra S., Hossain A., Brestic M., Skalicky M., Ondrisik P., Gitari H., Brahmachari K., Shankar T., Bhadra P., Palai J.B. (2021). Intercropping—A low input agricultural strategy for food and environmental security. Agronomy.

[4] Mrabet R. (2023). Sustainable agriculture for food and nutritional security. Sustainable Agriculture and the Environment.

[5] Joshi B.K., Shrestha H.K., Ayer D.K. (2023). Plant Breeding Strategies and Methods for Food Security: Review on the Technology. Emerging Solutions in Sustainable Food and Nutrition Security.

[6] Singh K. (2022). Agrobiodiversity, Status, and Conservation Strategies. Agro-Biodiversity and Agri-Ecosystem Management.

[7] Nishioka M. (2024). Changes in Temperature and CO_2_ in the Atmosphere at Various Latitudes. Curr. Res. Environ. Sci. Eco. Lett..

[8] Singh V. (2024). Forest Resources. Textbook of Environment and Ecology.

[9] Aziz G., Minallah N., Saeed A., Frnda J., Khan W. (2024). Remote sensing based forest cover classification using machine learning. Sci. Rep..

[10] Pawera L., Khomsan A., Zuhud E.A., Hunter D., Ickowitz A., Polesny Z. (2020). Wild food plants and trends in their use: From knowledge and perceptions to drivers of change in West Sumatra, Indonesia. Foods.

[11] Agúndez D., Lawali S., Mahamane A., Alía R., Soliño M. (2020). Farmers’ preferences for conservation and breeding programs of forestry food resources in Niger. Forests.

[12] Fusté-Forné F. (2022). Seasonality in food tourism: Wild foods in peripheral areas. Tour. Geogr..

[13] Gong R., Li X. (2025). The application progress and research trends of knowledge graphs and large language models in agriculture. Comput. Electron. Agric..

[14] Yan R., An P., Meng X., Li Y., Li D., Xu F., Dang D. (2025). A knowledge graph for crop diseases and pests in China. Sci. Data.

[15] Chamberlain J.L., Darr D., Meinhold K. (2020). Rediscovering the Contributions of Forests and Trees to Transition Global Food Systems. Forests.

[16] Lacuna-Richman C. (2006). The use of non-wood forest products by migrants in a new settlement: Experiences of a Visayan community in Palawan, Philippines. J. Ethnobiol. Ethnomed..

[17] Wang Y., Zhai Y., Ding Y., Zou Q. (2024). SBSM-Pro: Support bio-sequence machine for proteins. Sci. China Inf. Sci..

[18] Riyanto S., Sitanggang I.S., Djatna T., Atikah T.D. (2024). Plant-Disease Relation Model through BERT-BiLSTM-CRF Approach. Indones. J. Electr. Eng. Inform. (IJEEI).

[19] Hadjisofokelous C., Drakopoulos G., Sioutas S., Mylonas P. (2025). Discovering Fraudulent Card Transactions With Higher Order Graph Embeddings Over Neo4j. Proceedings of the IFIP International Conference on Artificial Intelligence Applications and Innovations.

